# Electric Belts and Pads

**Published:** 1891-10

**Authors:** J. S. Baker


### ELECTRIC BELTS AND PADS.

Of all the humbug devices by which quacks gull the public, none is more barefaced than the electric belt swindle. It is not very long ago that almost a quarter of the people in some sections of the country were wearing curious little disks about the size of a silver half dollar, composed of alternate smaller disks of zinc and copper. This combination was said to be a battery. It was attached to a string about the neck and was worn next the skin over the pit of the stomach, and it was supposed to cure dyspepsia, rheumatism, nervousness, and about every thing else.

As a proof that it was a genuine battery, the owner was instructed to place it on his tongue. Of course, the zinc and copper disks acted upon by the saliva, would cause a slight electric current to be generated, just as in the familiar experiment at school when the boys would place a piece of zinc above and a silver half dollar below the tongue in order to feel the galvanic current passing through that organ. Having felt this current in his sensitive tongue, the gullible patient was told that the same thing, constantly passing into his body from the surface, was a sure panacea for his ills,—and his fifty cents was quickly forthcoming.

But none but the most ignorant could for a moment suppose that the insignificant and imperceptible current generated by the action of the perspiration on the zinc and copper, would produce as much effect on a disease as the infinitely stronger and more evident current produced by rubbing the back of the family cat,—and the latter operation would be a far more sensible therapeutic measure.

We class all such things with the magnetic inhalers, hair brushes, caps, soles, etc., as being expensive toys at the best.

Charcot experimented with magnets, and thought he obtained wonderful results by their use, until, having used a wooden dummy in the shape of a magnet, and getting the same effects, he came to the conclusion that the whole thing was due to suggestion.

It is much the same with all the various remedial appliances above noted. If the patient is in need of electrical treatment it should be given by a medical man who thoroughly understands it, just as you would employ only an educated physician in any event of sickness or injury.—J. S. Baker, M. D., *in Healthy Home.*

